# A Simple and Efficient Magnesium Hydroxide Modification Strategy for Flame-Retardancy Epoxy Resin

**DOI:** 10.3390/polym16111471

**Published:** 2024-05-22

**Authors:** Linan Dun, Zeen Ouyang, Qihao Sun, Xiaoju Yue, Guodong Wu, Bohan Li, Weidong Kang, Yuanhao Wang

**Affiliations:** 1 College of Materials Science and Engineering, Northeastern University, Shenyang 110004, China; 2110204@stu.neu.edu.cn (L.D.); qihaosun@stumail.neu.edu.cn (Q.S.); 2 Hofmann Institute of Advanced Materials, Shenzhen Polytechnic, Shenzhen 518055, China; gycxd@hotmail.com (Z.O.); yuexiaoju@utibet.edu.cn (X.Y.); wuguodong@stu.xju.edu.cn (G.W.); libohanerick@163.com (B.L.); 3 Jinxi Industries Group Co., Ltd., Taiyuan 030000, China; 18734868028@163.com

**Keywords:** magnesium hydroxide, functional modification, epoxy resin, flame retardancy

## Abstract

Magnesium hydroxide, as a green inorganic flame-retardancy additive, has been widely used in polymer flame retardancy. However, magnesium hydroxide is difficult to disperse with epoxy resin (EP), and its flame-retardancy performance is poor, so it is difficult to use in flame-retardant epoxy resin. In this study, an efficient magnesium hydroxide-based flame retardant (MH@PPAC) was prepared by surface modification of 2-(diphenyl phosphine) benzoic acid (PPAC) using a simple method. The effect of MH@PPAC on the flame-retardancy properties for epoxy resins was investigated, and the flame-retardancy mechanism was studied. The results show that 5 wt% MH@PPAC can increase the limiting oxygen index for EP from 24.1% to 38.9%, achieving a V-0 rating. At the same time, compared to EP, the peak heat release rate, peak smoke production rate, total smoke production rate, and peak CO generation rate for EP/5 wt% MH@PPAC composite material decreased by 53%, 45%, 51.85%, and 53.13% respectively. The cooperative effect for PPAC and MH promotes the formation of a continuous and dense char layer during the combustion process for the EP-blend material, significantly reducing the exchange for heat and combustible gases, and effectively hindering the combustion process. Additionally, the surface modification of PPAC enhances the dispersion of MH in the EP matrix, endowing EP with superior mechanical properties that meet practical application requirements, thereby expanding the application scope for flame-retardant EP-blend materials.

## 1. Introduction

Epoxy resin (EP), as a typical thermosetting polymer material, has been widely used in the construction [1,2,3], electronics [4,5,6], and aerospace [7,8,9] fields due to its excellent thermal stability [10,11,12], electrical insulation [13,14], chemical resistance, mechanical properties, and processability [15,16,17]. However, like other polymer materials, EP is flammable, accompanied by a large number of harmful gases and smoke in combustion, which poses a great threat to human life and property safety. Therefore, there is still an urgent need to adopt a suitable processing method for manufacturing excellent flame-retardancy properties for EP materials to meet the requirements for various fields.

In recent years, inorganic flame retardants represented by magnesium hydroxide (MH) have gradually attracted people’s research interest due to their low environmental impact. MH has a high thermal decomposition temperature, is non-toxic and low-cost, and has an excellent smoke suppression effect, so it is widely used in the field for flame retardancy for polymers [18,19,20,21]. On the one hand, when MH is decomposed by heat, water vapor can be generated to dilute the combustible material in the gas phase generated by polymer degradation. On the other hand, MgO is formed during the decomposition process for MH, which can participate in the formation of a barrier carbon layer [22]. Lv [23] modified MH with amino-trimethylphosphonic acid and melamine to prepare a phosphorous nitrogen co-flame retardant (AM-MEL), which was combined with ammonium polyphosphate and incorporated into epoxy resin. When 5 wt% AM-MEL and 15 wt% APP are added, EP/AM-MEL/APP can achieve a limit oxygen index value of 32.0% and reach the vertical combustion test (UL-94) V-0 rating. Compared with pure EP, the peak heat release rate, total heat release rate, smoke production rate, and CO production rate were reduced by 88.0%, 70.0%, 81.5%, and 87.3%, respectively. Zhang [24] synthesized 2,4,6-tris(4-boronic-2-thiophene)-1,3,5-triazine (3TT-3BA) to cooperate with MH flame-retardant epoxy resin. The results show that the combination of 3TT-3BA and MH can significantly improve the thermal stability and flame retardancy of EP. When the ratio of 3TT-3BA and MH is 1:1, the limiting oxygen index for the composite can be increased to 32.5% and reach V-0 level using 20 wt% flame retardants. However, although MH can achieve a good smoke suppression effect, it cannot meet the demand for flame-retardancy properties for materials. Therefore, in order to obtain good flame-retardancy properties, MH needs to be modified and combined with other flame retardants. In addition, MH tends to agglomerate into larger particles in the resin matrix, deteriorating the mechanical and flame-retardancy properties for the material, which hinders the application of MH in EP flame retardancy.

Phosphorous-containing flame retardants have high efficiency and less toxic gas and smoke release, and have both condensing phase and gas phase flame-retardancy effects [25,26], so they have been widely developed to achieve flame-retardancy properties for polymer materials. In addition, organic flame retardants can be designed according to the need; for example, those containing carboxyl and hydroxyl groups of flame retardants have been developed and applied. By designing a functionalized organophosphorus flame-retardant monomer for functionalization of inorganic particles, good flame-retardancy performance can be achieved, and the dispersion of inorganic particles in the resin matrix can be improved. In this work, MH was modified with 2-(diphenyl phosphine) benzoic acid to prepare a novel, inexpensive, and environmentally friendly flame-retardant functionalized magnesium hydroxide (MH@PPAC). The effect of MH@PPAC on the fire safety of epoxy resin was studied by introducing MH@PPAC into the epoxy resin through the oxygen index, vertical combustion test, and cone calorimetry test. In addition, scanning electron microscopy (SEM), Fourier transform infrared analysis, and Raman spectra for the char layer were used to study the microstructure and chemical composition after combustion. 

## 2. Experimental

### 2.1. Materials

EP (E44) was purchased from Jinan Baorui Resin Chemical Co., Ltd. (China, Jinan). 4,4′-methylenedianiline (DDM), ethanol (99.7%), and 2-(diphenylphosphino)benzoic acid (PPAC) were purchased from Shanghai McLean Biochemical Technology Co., Ltd. (Shanghai, China) Magnesium hydroxide (MH) powder was purchased from Jiangsu Zehui Magnesium Based New Material Technology Co., Ltd. (Wuxi, China)

### 2.2. Preparation of the MH@PPAC

**Scheme 1:**
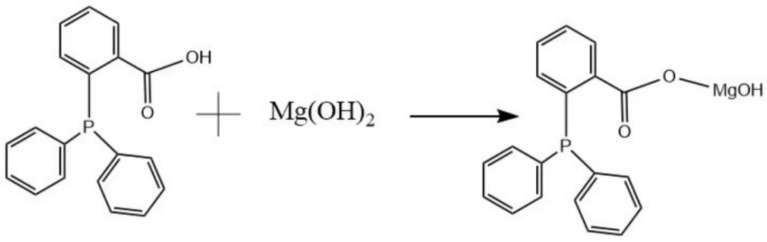
The synthetic route of MH@PPAC.

MH (1.45 g, 25.0 mmol) and 150 mL of ethanol were poured into a three-necked flask under mechanical stirring and stirred until completely dispersed. The PPAC (8.4234 g, 27.5 mmol) was dissolved in 150 mL of ethanol into a constant pressure dropping funnel and then slowly dripped into the three-necked flask. Subsequently, the mixture was heated to reflux and stirred for eight hours. At the conclusion of the reaction, the residual PPAC present in the solution was initially removed by filtration and then the filter cake was washed three times with ethanol. Subsequently, the product was subjected to vacuum drying at 80 °C for 12 h in order to obtain MH@PPAC powder. The solvent and PPAC in the filtrate were separated by distillation under reduced pressure in order to recover ethanol and unreacted PP AC. The specific reaction process is shown in Scheme 1.

### 2.3. Preparation for EP Flame-Retardant Composites

**Scheme 2:**
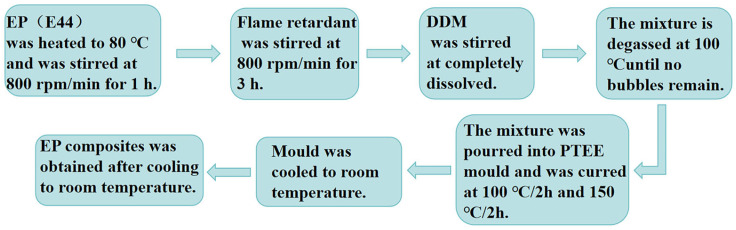
The preparation process for the EP flame-retardant composites.

A series of EP blends containing MH or MH@PPAC were prepared by a simple blending method, and the specific compositions are shown in Table 1. The specific preparation process for the composites is shown in Scheme 2.

### 2.4. Characterization

The characteristic functional groups of MH, PPAC, and MH@PPAC were detected using a PE Frontier model FTIR spectrometer in the range of 400~4000 cm^−1^, with potassium bromide serving as the substrate. XPS (via a Thermo Scientific spectrometer) was conducted using K-alpha radiation. High resolution mass spectrometry (HRMS) was used to test the relative molecular mass of MH@PPAC, using an Agilent 1260-6530A. In order to observe the thermal decomposition behavior of MH, PPAC, and MH@PPAC, TGA tests were conducted on a TGA550 (Netzsch, Selb, Germany) under a N_2_ atmosphere with a heating rate of 10 °C/min and a temperature ranging from 30 to 700 °C. The limiting oxygen index (LOI) values for epoxy polymer (EP) and EP-blend materials were measured using an oxygen index apparatus (FTT, Derby, UK), in compliance with ASTM D2863-77 standards. The dimensions for the test samples were 130 × 6.5 × 3.2 mm^3^. The UL-94 test was conducted in a combustion chamber (PHINIX, Chongqing, China) according to the ASTM D3801 standard, with test details specifying sample dimensions of 130 × 13 × 3.2 mm^3^. In accordance with ISO 5660 standards, samples with dimensions of 100 × 100 × 4 mm^3^ were placed in a cone calorimeter (FTT0007, Derby, UK) for cone calorimetry testing under an external heat flux of 50 kW/m^2^. A thermogravimetric analyzer (Perkin Elmer, Shelton, CT, USA, TGA4000) coupled with Fourier Transform Infrared Spectroscopy (Thermo, Norristown, PA, USA, Nicolet iS50) was utilized to characterize the evolved gaseous products from EP and EP-blend materials. The samples were heated from room temperature to 600 °C at a heating rate of 10 °C/min under a N_2_ atmosphere. The tensile properties for EP and EP-blend materials were tested on a CMT6103 device (MTS CMT6103, Eden Prairie, MN, USA) at a testing speed of 2 mm/min, in compliance with ASTM D 638 standards. The fracture surface morphology and char residue morphology after CCT tests were observed using a scanning electron microscope (SEM) on a JEOL/JSM-IT800 (SHL, Tokyo, Japan), with a working voltage of 10 kV.

## 3. Results and Discussion

### 3.1. MH@PPAC Characterization

#### 3.1.1. Structure Characterization of MH@PPAC

**Figure 1:**
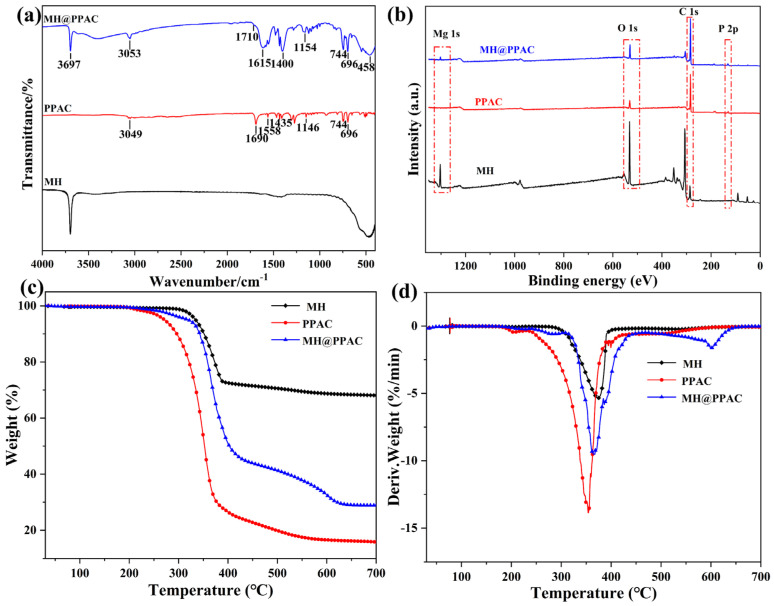
(**a**) FT-IR, (**b**) XPS, (**c**) TG, and (**d**) DTG for MH, PPAC, and MH@PPAC.

The structures of MH, PPAC, and MH@PPAC were characterized by FTIR spectroscopy, and the results are shown in Figure 1a. In MH@PPAC, the strong absorption peak at 3697 cm^−1^ is attributed to the characteristic stretching vibration of -OH. The peaks at 3053 cm^−1^ and 1615 cm^−1^ correspond to the C-H and C=C stretching vibrations in the benzene ring, respectively. Additionally, in MH@PPAC, the tensile vibration peak of C-P is at 1154 cm^−1^, the C=O bond tensile peak is at 1710 cm^−1^, the C-O bond tensile peak is at 1400 cm^−1^, the bending vibration region outside the C-H plane is at 696 cm^−1^ and 744 cm^−1^, and the O-Mg bond tensile peak is at 458 cm^−1^. The positions of the characteristic peaks in MH@PPAC were found to be shifted to different degrees in comparison to PPAC. A comparison of MH and MH@PPAC using FT-IR showed significant differences between them, which confirms the successful synthesis of the MH@PPAC flame retardant.

To further demonstrate the successful synthesis of flame retardants, the chemical composition of MH@PPAC was detected by X-ray photoelectron spectroscopy (XPS), and the results are shown in Figure 1b. In the XPS spectrum for MH@PPAC, characteristic peaks at 1304.2 eV, 531.1 eV, 285.2 eV, and 132.8 eV can be clearly observed, which are attributed to the Mg1s, O1s, C 1s, and P2p spectral bands, respectively. Upon comparison with MH, it can be noted that in MH@PPAC, a new characteristic P2p spectral band appears at 132.8 eV, which aligns closely with the position of P2p in PPAC. These results further confirmed the successful synthesis of MH@PPAC products. Following XPS analysis, the elemental composition of each element in MH@PPAC was determined, with the following results: P: 8.96%, C: 65.90%, O: 13.87%, and Mg: 6.94%.

**Figure 2:**
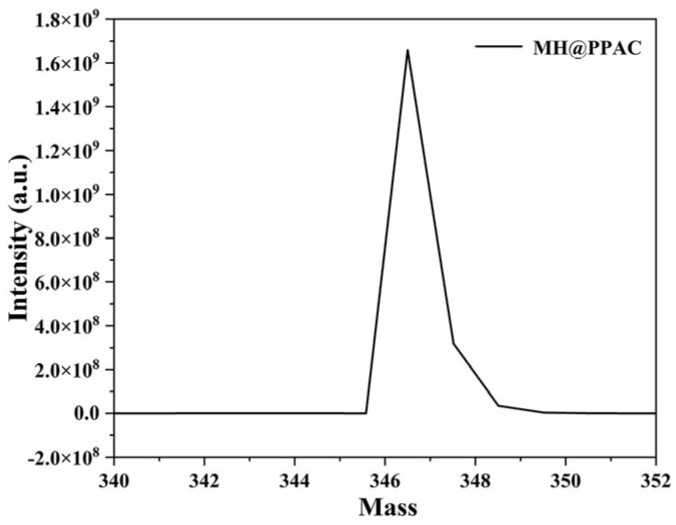
HRMS for MH@PPAC.

In order to further verify the chemical structure of MH@PPAC, HRMS analysis was carried out, and the experimental results are shown in Figure 2. The results demonstrated that the experimental relative molecular mass of MH@PPAC was 346.5990, which was consistent with the expected theoretical calculated value of the molecular formula (346.598762) and provided a decisive basis for the structural identification of the compound.

To analyze the thermal stability of MH, PPAC, and MH@PPAC, TGA and DTG measurements were conducted under N_2_ conditions, and the results are shown in Figure 1c,d and Table 2. It can be seen from the thermogravimetric data that the thermal decomposition temperatures of PPAC and MH@PPAAC are lower than that of MH, which can be attributed to the presence of a large number of oxygen-containing functional groups in PPAC. The weight loss process of MH@PPAC is a two-stage degradation process, with the initial thermal decomposition temperature (T-5%) and the maximum weight loss (T_max_) being 316 °C and 356 °C, respectively. Meanwhile, the thermal stability of MH@PPAC is better than that of PPAC. In addition, the DTG curve given in Figure 1d shows that the maximum weight loss rate (R_max_) of MH is 42.93%/°C at 383 °C, while the R_max_ values of PPAC and MH@PPAC at 367 °C and 356 °C are 33.53% and 19.70%/°C, respectively. The reduced thermal stability of MH@PPAC compared to MH is due to the introduction of a large number of organic structures.

#### 3.1.2. Morphology Analysis

**Figure 3:**
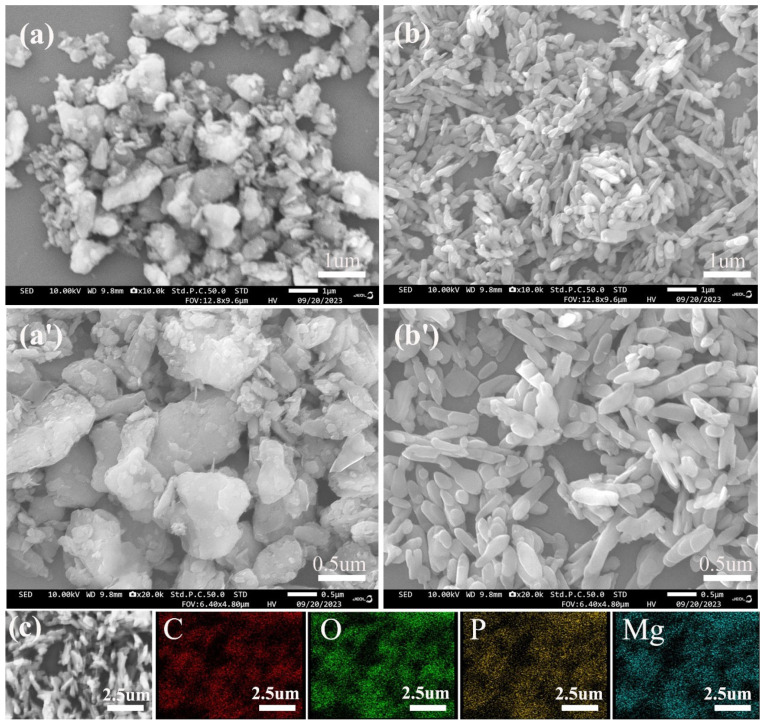
SEM images for MH (**a**,**a’**) and MH@PPAC (**b**,**b’**,**c**), and elemental mapping of MH@PPAC (C, O, P, Mg).

Figure 3 shows SEM images for MH and MH@PPAC. The morphology of MH is irregular and blocky, with a rough surface, strong surface effect, and particle agglomeration. However, the morphology of MH@PPAC shows smooth rod-like bodies with uniformly dispersed particles. Upon observation, it can also be noted that the particle size of MH noticeably decreases after the modification, which will be beneficial for the dispersion for MH@PPAC in the EP. Therefore, this will minimize the potential adverse effects of polymerizing MH particles on their mechanical properties. In addition, from the EDS surface scanning results of MH@PPAC (C), it can be observed that C, O, Mg, and P are uniformly distributed on its surface, and the appearance of C and P further confirms the successful synthesis of MH@PPAC hybrid products.

### 3.2. Thermal Stability of the EP Blends

**Figure 4:**
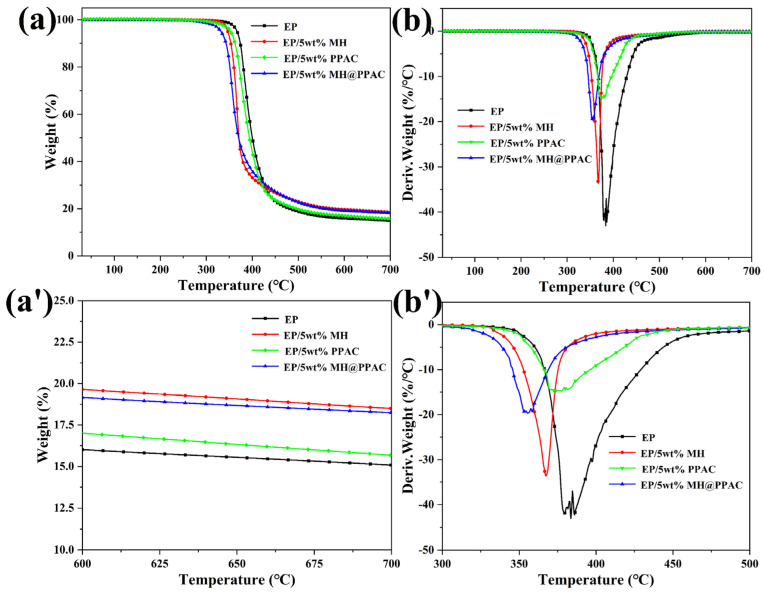
TGA (**a**,**a’**) and DTG (**b**,**b’**) curves for EP and EP blends under nitrogen atmosphere.

The thermal stability of EP and its composites was evaluated by thermogravimetric analysis in a nitrogen atmosphere, and the results are shown in Figure 4 and Table 3. From Figure 4, it can be seen that the thermal degradation of EP and its composites is a one-step degradation process. However, in contrast to EP, the inclusion of MH, PPAC, and MH@PPAC reduces the initial degradation temperature of EP from 367 °C to 346 °C, 355 °C, and 333 °C, respectively. This can be attributed to the metal oxides produced by the pyrolysis of MH, and the metal oxides and phosphide produced by MH@PPAC pyrolysis, which can catalyze the early degradation of the EP matrix and the occurrence of cross-linking reactions. The pyrolysis products of MH, PPAC, and MH@PPAC can participate in the formation of a carbon layer and catalyze the carbonization of the EP matrix, which increases the char yield of EP. In addition, it can be further observed from the DTG curve that the maximum mass loss rate of MH, PPAC, and MH@PPAC composites is much lower than that of the original EP. This can be attributed to the higher char yield during pyrolysis, which facilitates the formation of a physical barrier that prevents the heat and pyrolysis product transfer process, thereby inhibiting the degradation of EP [27,28]. 

### 3.3. Mechanical Performance

**Figure 5:**
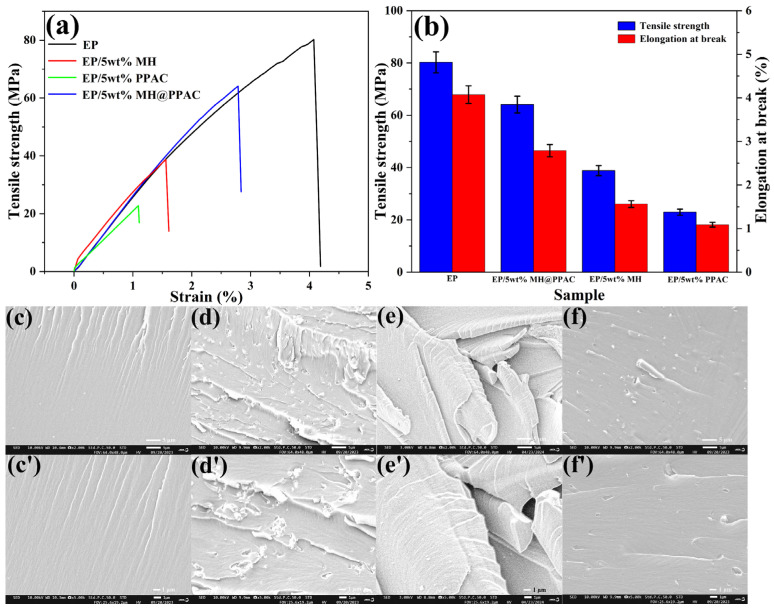
EP, EP/5 wt% MH, EP/5 wt% PPAC, and EP/5 wt% MH@PPAC tensile data (**a**,**b**) and SEM images of brittle fracture of the EP (**c**,**c’**), EP/5 wt% MH (**d**,**d’**), EP/5 wt% PPAC (**e**,**e’**), and EP/5 wt% MH@PPAC (**f**,**f’**).

Figure 5 shows the strain–stress curve, which reflects the mechanical properties of EP blends. The tensile strength of EP is 80.246 MPa. Unfortunately, the tensile strength of EP/5 wt% MH@PPAC is only 64.075 MPa, which is about 20.15% lower than that of pure EP, and significantly higher than that of EP/5 wt% MH (38.837 MPa) and EP/5 wt% PPAC (22.946 MPa). In order to study the dispersion and interfacial interaction between additives and EP, the cross-sections of EP and its composites were analyzed by SEM. As shown in Figure 5c,c’, the EP cross-section is smooth with only a few gullies. On the contrary, the EP/5 wt% MH surface shown in Figure 5d,d’ shows a large number of agglomerated particles and many holes and folds, which can be attributed to the aggregation of MH in the EP matrix to form larger particles that are pulled out when the material is fractured. The presence of a large number of agglomerated particles in the EP/5 wt% MH matrix is also an important reason for the deterioration for its mechanical properties. The cross-section of EP/5 wt% PPAC (Figure 5e,e’) exhibits a considerable number of flap-like structures, which are also a significant contributor to the deterioration of the mechanical properties of the composites. However, the cross-section of EP/5 wt% MH@PPAC (Figure 5f,f’) also shows a gully structure, with only a few smaller particles present. Compared with EP/5 wt% MH, the cross-section morphology is smoother, there are fewer pores, and the additive particles are smaller, which proves that the dispersion of magnesium hydroxide modified by PPAC in EP is greatly improved, and it has good compatibility with the EP matrix. This is the main reason why its mechanical properties are better than those of EP/5 wt% MH. 

### 3.4. Flame-Retardancy Behavior for EP-Blend Materials

The limiting oxygen index (LOI) and vertical combustion test (UL-94) are important indexes used to evaluate the flame retardancy of materials. In this study, LOI and the vertical combustion test were used to study the flame retardancy of EP and its composites. As shown in Table 3, the LOI value of EP was only 24.5%, while the LOI value of EP increased to 26.90% after the addition for MH. Unfortunately, EP and EP/5 wt% MH did not pass any UL-94 ratings. However, with the addition for 5 wt% MH@PPAC, the LOI value of EP can reach a higher value of 38.9% and its combustion level can reach V-0. With the exception of EP, the other two composites showed no obvious signs of being ignited.

**Figure 6:**
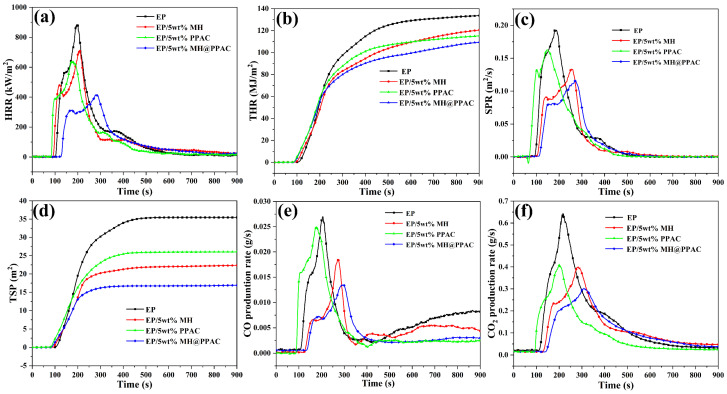
(**a**) HRR, (**b**) THR, SPR (**c**), TSP (**d**), PCOP (**e**), and PCO_2_P (**f**) curves for EP and its composites.

The cone calorimetric test (CCT) is generally regarded as an important standard for evaluating the combustion performance of polymer materials, and can be used to monitor the heat and smoke release and other parameters in the whole combustion process in real time. In this study, the flame-retardancy performance of EP and its composites was analyzed, and the specific data are shown in Figure 6. As shown in Figure 6a, which shows the heat release rate (HRR) curves for EP and its composites, the peak heat release rate (PHRR) of EP is reached at about 198 s into combustion, with a value of 883 kW/m^2^. However, the PHRR for EP/5 wt% MH and EP/5 wt% MH@PPAC is significantly delayed. Conversely, the PHRR for EP/5 wt% PPAC is advanced compared to that of EP. The HRR for EP shows a bimodal phenomenon after the addition of MH and MH@PPAC due to the suppression of the generation and volatilization of combustible gas during heating [28]. Meanwhile, PHRR values for EP/5 wt% MH, EP/5 wt% PPAC, and EP/5 wt% MH@PPAC were reduced to 710 kW/m^2^, 637 kW/m^2^, and 415 kW/m^2^, respectively, which were 19.59%, 27.86%, and 53.00% lower than that of EP. In addition, the total heat release (THR) of EP was significantly reduced after the addition of MH, PPAC, and MH@PPAC, which means that the composite poses less of a threat to humans in a real fire. Importantly, MH@PPAC exhibits lower PHRR and THR values than MH and PPAC with the same content, indicating higher flame retardancy.

In a fire, smoke and gas production is even more harmful to human life and property safety than the release of heat. According to statistics, more than half of the deaths in house fires are caused by inhalation of toxic smoke [29]. Figure 6c,d shows the smoke production rate (SPR) for EP and its composites and the total rate of smoke production (TSP). Figure 6c,d shows that EP releases higher values of SPR and TSP during combustion. On the contrary, SPR and TSP values of EP/5 wt% MH, EP/5 wt% PPAC, and EP/5 wt% MH@PPAC were significantly decreased, and the decrease in that of EP/5 wt% MH@PPAC was more obvious, at up to 45%. Additionally, CO and high-concentration CO_2_ produced by fires are also among the important causes of human casualties. The information in Figure 6e,f shows that MH, PPAC, and MH@PPAC can each inhibit the release of CO and CO_2_ during EP combustion, and MH@PPAC is more effective. As expected, the peak CO production rate (PCOP) and peak CO_2_ production rate (PCO_2_P) for EP/5 wt% MH@PPAC were 0.013 g/s and 0.30 g/s, respectively, which were significantly reduced, by 51.85% and 53.13%, compared with those of pure EP (0.027 g/s and 0.64 g/s). The simultaneous decrease of PCOP and PCO_2_P indicates that the addition of flame retardants can catalyze EP to form a dense char layer to inhibit further decomposition of the underlying substrate, thus achieving smoke suppression and toxicity reduction.

**Figure 7:**
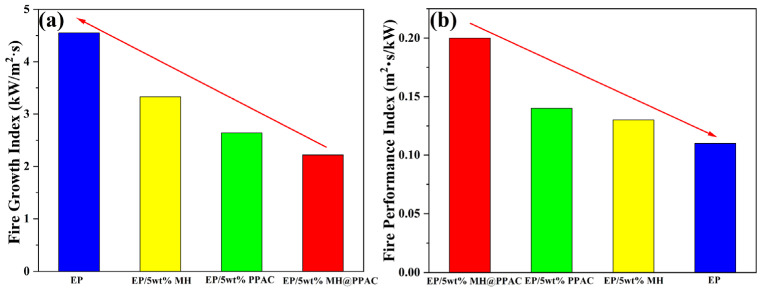
(**a**) Fire growth index and (**b**) fire performance index for EP and its composites.

Typically, the fire growth index (FGI) and fire performance index (FPI) are considered to be important parameters for evaluating the fire safety factor [30]. FPI = TTI/PHRR, which can well characterize the potential hazard of materials in fire; and FGI = PHRR/T, which reflects the spread of the fire after the material catches fire. In general, if the material has a high safety level, it needs to meet the two conditions of having a high fire performance index and a low fire growth index. As shown in Figure 7, the incorporation of MH@PPAC can significantly improve the FPI value compared to that of pure EP, indicating a lower potential hazard of the fire for the EP blend. At the same time, the significant decrease in FGI indicates a longer time to reach PHRR, which means that MH@PPAC is favorable for slowing down flame spread during EP combustion [31]. EP/5 wt% MH@PPAC exhibits the highest FPI (0.20) and the lowest FGI (2.22), further demonstrating its excellent fire safety. In order to ensure the accuracy of the CTT experimental results, three replicate experiments were conducted, and the specific experimental data are listed in Table 4. Table 5 presents a comparison of the experimental results of this study with those reported in the references. The findings indicate that MH@PPAC exhibits enhanced flame retardancy, positioning it at the forefront of this field. Furthermore, the ageing of polymer materials severely constrains its development. Consequently, a second test was conducted after the composites were stored at room temperature for a period of three months. The results demonstrated that the composites exhibited no discernible signs of significant ageing.

### 3.5. Flame-Retardancy Mechanism

#### 3.5.1. Analysis of the Char Residues

**Figure 8:**
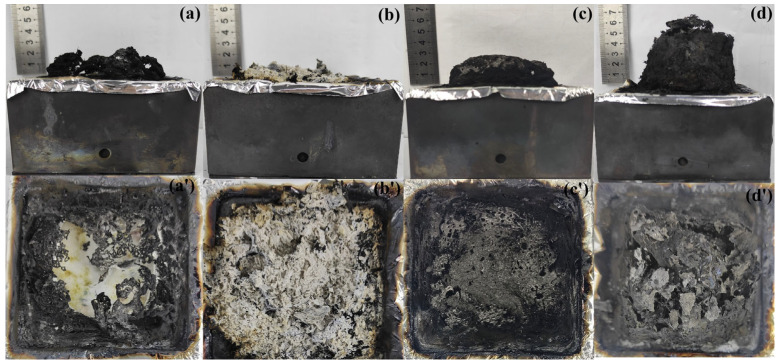
Digital images of (**a**,**a’**) EP, (**b**,**b’**) EP/5 wt% MH, (**c**,**c’**) EP/5 wt% PPAC, and (**d**,**d’**) EP/5 wt% MH@PPAC for charcoal residue from the cone calorimetry experiment.

**Figure 9:**
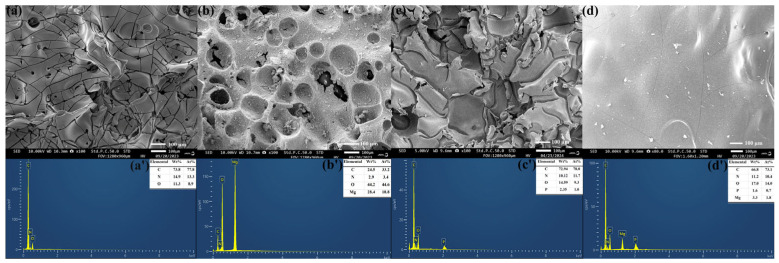
Scanning electron microscope images and elemental distribution of (**a**,**a’**) EP, (**b**,**b’**) EP/5 wt% MH, (**c**,**c’**) EP/5 wt% PPAC, and (**d**,**d’**) EP/5 wt% MH@PPAC of charcoal slag from the conical calorimetry experiment.

Studying the digital photos and microstructure of the char residues is helpful to understand the condensed phase mechanism of EP blends. The digital photos and SEM images of the sample surface after the CCT test are shown in Figure 8 and Figure 9. It can be seen from Figure 8a that pure EP is almost burned out after burning, exposing the bottom aluminum foil. Furthermore, EP containing MH, PPAC, and MH@PPAC formed a large amount of char residue after combustion, and the char exhibited varying degrees of expansion. The SEM images of the char layer reveal that EP, EP/5 wt% MH, EP/5 wt% PPAC, and EP/5 wt% MH@PPAC present significantly different microstructures. As shown in Figure 9a, a large number of cracks appeared on the surface of the carbon slag after EP combustion, which can be attributed to the fact that a large amount of gas formed by EP combustion rushed out of the char layer and cracked it, thus accelerating the release of heat and pyrolysis products, resulting in the near burnout of EP [42]. After combustion, EP/5 wt% MH forms a porous char layer, which blocks the transfer of heat and gas and protects the substrate at the bottom. The residual carbon of EP/5 wt% PPAC exhibits a surface morphology that is similar to that of pure EP. Surprisingly, the char layer formed after combustion of EP/5 wt% MH@PPAC is significantly different from that of EP and EP/5 wt% MH, with a more complete and compact char layer. The dense carbon layer barrier prevents heat from entering the polymer to slow the polymer degradation rate and reduce the release of pyrolysis vapor products during combustion [43,44], which can be demonstrated by the significant reduction in THR and TSP.

As can be seen from the EDS data for the char layer in Figure 9, EP has three main elements, consisting of C, O, and N. In the EP blends containing MH and MH@PPAC, Mg and P elements are also contained in the residual carbon, besides the C, O, and N elements. For EP/5 wt% MH, the increase in O and Mg elements and the decrease in C elements indicate the formation of magnesium oxide during the combustion of the composite. For EP/5 wt% MH@PPAC, the carbon layer not only contains magnesium oxide, but is also rich in the P element, indicating that cross-linked phosphate-containing compounds are formed in the combustion process of the composite, which improves the quality of the char layer. This is also an important reason why the microstructure of the char layer is significantly different from that of EP and EP/5 wt% MH.

**Figure 10:**
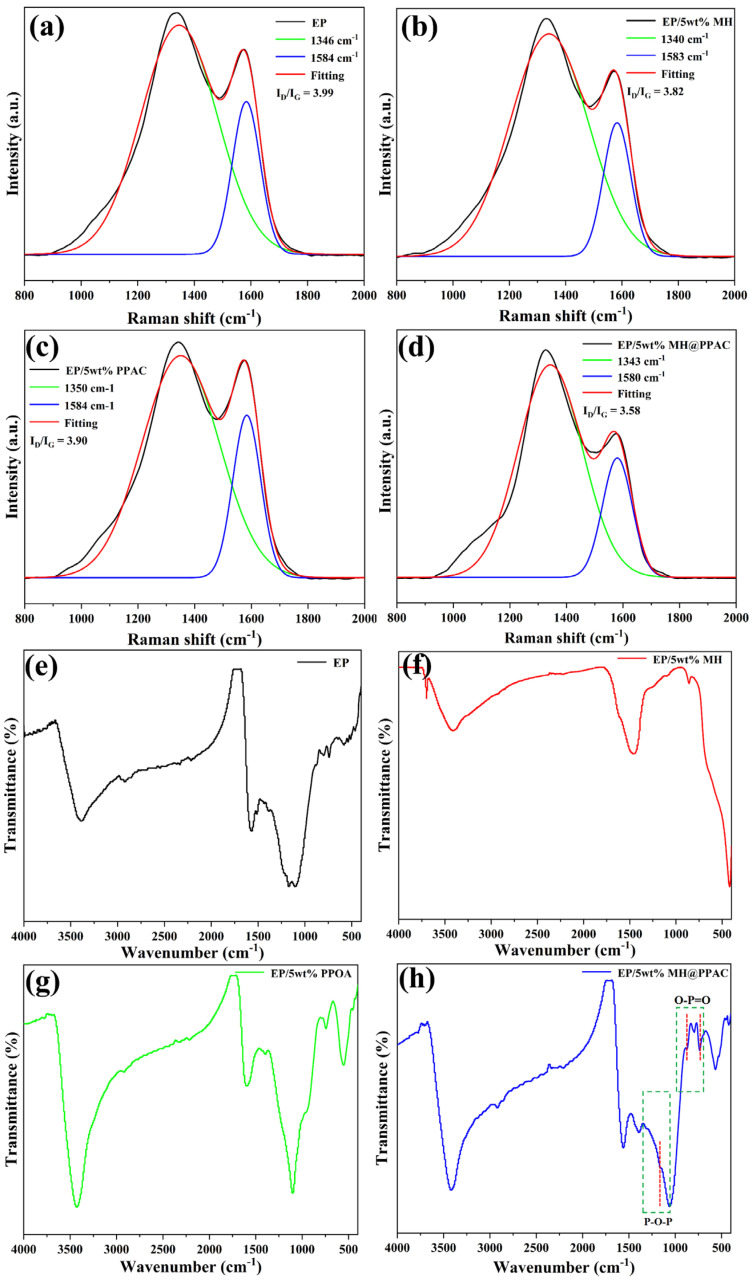
Raman shift of (**a**) EP, (**b**) EP/5 wt% MH, (**c**) EP/5 wt% PPAC, (**d**) EP/5 wt% MH@PPAC and FT-IR spectra, (**e**) EP, (**f**) EP/5 wt% MH, (**g**) EP/5 wt% PPAC, and (**h**) EP/5 wt% MH@PPAC of charcoal residue from conical calorimetry experiments.

As shown in Figure 10a–d, the Raman shift of the residue from the EP blends after the cone calorimeter experiment is depicted. The I_D_/I_G_ ratio (the ratio of the peak areas of the D band to the G band) is commonly used as an important basis for characterizing the degree of graphitization of the residual char. Generally, a smaller I_D_/I_G_ ratio indicates a higher graphitization degree of the carbon material and a better graphitized layer structure. As can be seen from Figure 10, I_D_/I_G_ of the char residue of EP is 3.99, while EP/5 wt% MH@PPAC shows the lowest I_D_/I_G_ (3.58) among all EP blends, indicating that the addition of MH@PPAC makes the composite form a denser char layer [45], which is consistent with the results from SEM. Therefore, it can be inferred that the cooperative catalytic carbonization effect between P and Mg leads to a greater quantity and quality of char, which acts as an effective physical barrier, enhancing the fire safety of the EP blends.

As shown in Figure 10e–h, the composition for the char residue was analyzed by FT-IR. Figure 10h presents the FTIR spectrum of the char residue from the EP/5 wt%MH@PPAC composite material. The characteristic absorption peaks at 732 cm^−1^ and 564 cm^−1^ correspond to the bending vibrations of O-P=O in PO_4_^3−^, and the characteristic absorption peak of P-O-P appears at 1061 cm^−1^. This demonstrates that the decomposition of MH@PPAC generates phosphorus-containing substances such as phosphoric acid and pyrophosphate [46], which effectively promote the carbonization and cross-linking of EP to form a dense carbon layer. This is also the reason why the yield and density of EP/5 wt% MH@PPAC char residual are better than those of EP/5 wt% MH and pure EP in the CCT test results. Therefore, the cooperative effect of MH and PPAC plays a crucial role in catalyzing the formation of a dense and expanded char layer.

#### 3.5.2. Gaseous Phase Analysis

**Figure 11:**
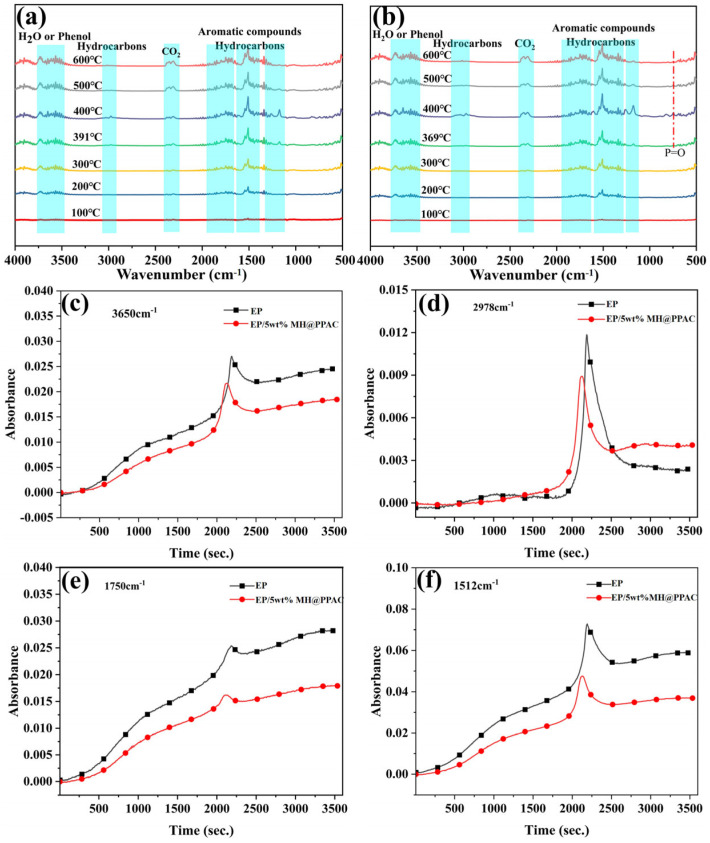
FT−IR spectra for pyrolysis products of EP (**a**) and EP/5 wt% MH@PPAC (**b**); the absorbance of typical pyrolysis products (**c**–**f**) for EP and EP/5 wt% MH@PPAC.

The volatilities of EP and its composites were further investigated by TGA-FTIR, and the results are shown in Figure 11. For the original EP, some characteristic absorption peaks of FT-IR were attributed to phenol and water (3650 cm^−1^), hydrocarbons (2978 cm^−1^), carbon dioxide (2348 cm^−1^ and 2312 cm^−1^), carbonyl compounds (1750 cm^−1^), and aromatic compounds (1512 cm^−1^) [47,48,49]. The main gaseous products of EP/5 wt% MH@PPAC are similar to those of EP, indicating that the flame retardancy does not change the types of degradation product. Additionally, the formation of phosphorous-containing products detected at 742 cm^−1^ after 369 °C indicates that P· radicals combine with O· radicals and is released into the gas phase during pyrolysis, which plays a role in gas phase quenching during combustion [50]. Interestingly, compared with the original EP, MH@PPAC shows a better effect in suppressing the release of hydrocarbons, carbonyl compounds, and aromatic compounds generated from the degradation of EP (Figure 11c–f), which means fewer organic compounds are involved in the combustion. This result indicates that MH@PPAC can effectively inhibit the gas generation from EP decomposition and convert it into a dense char layer, which explains well the increased char residue observed in CCT and TGA tests. The reduction in the absorbance of carbonyl compounds indicates that MH@PPAC inhibits the production of carcinogens [48,51]. The reduction in gaseous products such as aromatic compounds confirms that the release of smoke and toxic gases from EP has been effectively suppressed. In summary, MH@PPAC can serve as an effective gas phase flame retardant, achieving smoke suppression and toxicity reduction [52,53].

Based on the above analysis, the flame-retardancy mechanism of MH@PPAC on EP can be obtained. When the temperature reaches the ignition temperature, the EP/5 wt% MH@PPAC composite undergoes combustion and thermal degradation. On one hand, MH@PPAC is decomposed by heat to form MgO, and phosphoric acid and pyrophosphate can catalyze the formation of a stable carbon layer, preventing the thermal oxygen transfer and fuel supply and the release of pyrolysis gas. On the other hand, the thermal decomposition of PPAC also produces P· radicals that can combine with O· free radicals and are released into the gas phase, serving as gas phase free radical quenchers during the combustion process. Additionally, the non-combustible gases (such as NH_3_, CO_2_, and H_2_O) produced by the thermal decomposition of the EP-blend material dilute the combustible gases and oxygen on the matrix surface, which is beneficial to the formation of the char layer. Therefore, MH@PPAC can serve as a flame retardant simultaneously in both the gas and condensed phases, achieving efficient flame retardancy for EP.

## 4. Conclusions

In this study, MH@PPAC was designed and synthesized to enable the EP blend to have higher fire safety, which is reflected in low flammability, less smoke, and less toxic gas emissions during the combustion of the EP polymer. The addition of 5 wt% MH@PPAC to EP can increase the LOI value to 28.9%, and reduce the PHRR and TSP by 53% and 45%, respectively. Additionally, compared to pure EP, PCOP and PCO_2_P are significantly reduced by approximately 51.85% and 53.13%, respectively. Due to the cooperative effect of MH and PPAC, the formation of a continuous and highly expanded dense char layer in the EP blends is promoted, effectively hindering the processes of heat flow and flammable gas exchange. In addition to the barrier effect of the char layer, the phosphorus oxides produced by PPAC during combustion can interrupt the free radical chain reactions of combustion, which is another important step to ensure the fire safety of EP blends is improved. PPAC also significantly improves the dispersion of MH in the EP matrix, enhancing the mechanical properties of the EP blends. A simple strategy of modifying MH was used to prepare flame-retardant EP with excellent fire and smoke suppression properties, which provided a meaningful attempt at the development and application of MH in flame-retardant EP.

## Figures and Tables

**Table 1:** Formulations of EP blends.

Samples	EP (wt.%)	DDM (wt%)	MH (wt%)	PPAC (wt%)	MH@PPAC (wt%)
EP	82.0	18.0	0.0	0.0	0.0
EP/5 wt% MH	77.4	17.6	5.0	0.0	0.0
EP/5 wt% PPAC	77.4	17.6	0	5.0	0
EP/5 wt% MH@PPAC	77.4	17.6	0.0	0.0	5.0

**Table 2:** Thermal data of MH, PPAC, and MH@PPAC in N_2_.

Samples	T-5% ^a^ (°C)	T_max_ ^b^ (°C)	Char ^c^ (%)
MH	334	383	68.12
PPAC	273	367	16.08
MH@PPAC	316	356	28.98

^a^ Temperature at 5 wt.% weight loss. ^b^ Temperature at maximum mass loss rate. ^c^ Residue at 700 °C.

**Table 3:** TGA data for EP and EP blends.

Samples	T_-5%_ ^a^ (°C)	T_-50%_^b^ (°C)	T_max_ ^c^ (°C)	Char ^d^ (%)
EP	367	401	384	14.97
EP/5 wt% MH	346	370	368	18.50
EP/5 wt% PPAC	355	395	375	15.51
EP/5 wt% MH@PPAC	333	369	355	18.90

^a^ Temperature at 5 wt% weight loss. ^b^ Temperature at 50 wt% weight loss. ^c^ Temperature at maximum mass loss rate. ^d^ Residue at 700 °C.

**Table 4:** Selected data from the cone calorimetry experiment.

Samples	EP	EP/5 wt% MH	EP/5 wt% PPAC	EP/5 wt% MH@PPAC
LOI (%)	24.5 ± 0.20	26.9 ± 0.30	26.3 ± 0.21	38.9 ± 0.26
TTI (s)	102 ± 3	92 ± 2	83 ± 3	85 ± 2
t PHRR (s)	198 ± 3	210 ± 2	241 ± 5	283 ± 2
PHRR (kW/m^2^·S)	883 ± 15	710 ± 21	637 ± 16	415 ± 19
FGI (kW/(m^2^·S))	4.55 ± 0.31	3.33 ± 0.23	2.64 ± 0.32	2.22 ± 0.16
FPI (S·m^2^/kW)	0.11 ± 0.06	0.13 ± 0.09	0.14 ± 0.10	0.20 ± 0.12
PSPR (m^2^/s)	0.20 ± 0.10	0.13 ± 0.08	0.16 ± 0.03	0.11 ± 0.07
TSP (m^2^)	35.4 ± 5.61	22.4 ± 3.91	26.10 ± 2.36	16.8 ± 2.30
PCOP (g/s)	0.027 ± 0.005	0.018 ± 0.003	0.025 ± 0.009	0.013 ± 0.003
PCO_2_P (g/s)	0.64 ± 0.12	0.40 ± 0.09	0.41 ± 0.008	0.30 ± 0.12
UL-94	NR	NR	NR	V-0

**Table 5:** Comparison of experimental results of other references with the experimental results of this work.

Reference	LOI (%)	PHRR (kW/m^2^·S)	THR (MJ/m^2^)	TSP (m^2^)	UL-94
[32]	31.5	535	67	38.9	V-1
[33]	33.0	471.3	69.5	34.4	V-1
[34]	30.0	1059.1	66.8	8.20	V-1
[35]	34.2	557	90.1	32.02	V-0
[36]	35.2	658	51.3	26.96	V-0
[37]	36.7	507.2	25.9	-	V-0
[38]	30.3	696.12	79.09	26.14	V-0
[39]	36.1	385.4	53.1	9.9	V-0
[40]	35.2	625.5	62.9	-	V-0
[41]	27.9	450.65	124.98	21.87	V-0
This work	38.9	415	109.40	16.8	V-0

## Data Availability

Data are contained within the article.
